# App-based mindfulness meditation reduces stress in novice meditators: a randomized controlled trial of headspace using ecological momentary assessment

**DOI:** 10.1093/abm/kaaf025

**Published:** 2025-04-21

**Authors:** Matthew J Zawadzki, Zoltan A Torok, Mercedes Peña, Larisa Gavrilova

**Affiliations:** Department of Psychological Sciences, University of California, Merced, CA 95353, United States; Health Sciences Research Institute, University of California, Merced, CA 95343, United States; Department of Psychological Sciences, University of California, Merced, CA 95353, United States; Health Sciences Research Institute, University of California, Merced, CA 95343, United States; Department of Psychological Sciences, University of California, Merced, CA 95353, United States; Health Sciences Research Institute, University of California, Merced, CA 95343, United States; Department of Psychological Sciences, University of California, Merced, CA 95353, United States

**Keywords:** Headspace, mindfulness, mHealth, digital health, stress

## Abstract

**Background:**

App-based mindfulness meditation programs have shown mixed effects in reducing stress levels. These studies have typically relied on limited assessments of dimensions of stress and on pre-post designs to detect effects.

**Purpose:**

This randomized controlled trial examined the effect of the mindfulness meditation app Headspace on reducing subjective stress, stressor appraisals, perceived coping, and perseverative cognitions. It tested stress-reducing effects in everyday life throughout an eight-week intervention period.

**Method:**

Non-faculty employees (*n *= 138; age *M* = 38.19; 75.36% female; 54.5% White, 27.54% Hispanic; 51.45% with a professional degree) from a university in California’s Central Valley were randomized into either the Headspace condition (instructed to complete 10 minutes of meditation daily) or wait-list (inactive) control group. Participants completed ecological momentary assessments of stress five times a day for four consecutive days at baseline, at two and five weeks after randomization (mid-intervention), and at eight weeks post-randomization (post-intervention), resulting in 6260 observations of stress dimensions.

**Results:**

Hierarchical linear models were used to test the interaction of condition by time, revealing significant effects for subjective stress, perceived coping, and perseverative cognitions. By week 2, compared to the baseline, participants in the Headspace condition reported less subjective stress and perseverative cognitions, and by week 5 reported more perceived coping. These effects persisted through week 8. No changes were observed for stressor appraisal. Participants in the control condition reported increases in subjective stress and perseverative cognitions, and decreases in coping, throughout the intervention period.

**Discussion:**

Headspace was effective at reducing stress in a high-stress environment. Findings suggest the potential for relatively quick and sustained gains in stress benefits from meditation practice that may help practitioners develop their future programs.

## Teaser

People new to meditation who used Headspace (a mindfulness meditation app) reported a decrease in feelings of stress, a decrease in repetitive thinking, and an increase in perceptions of one’s ability to confront stress.

In-person mindfulness meditation, including the popular Mindfulness-Based Stress Reduction program (MBSR) program, has been shown to reduce stress and improve physical and psychological health outcomes.^1^ More recently, mindfulness meditation training has shifted online, particularly through smartphone applications. These apps provide a different experience than in-person mindfulness meditation, necessitating the need to test whether they can effectively improve outcomes, particularly for those new to meditation. Current research suggests mixed results to their efficacy.^2^ For novice meditators, app-based mindfulness may be challenging as it is primarily an individual experience with one-way instruction. Yet, the apps can be personalized to a degree, including when the meditation is done and for how long, which may allow the novice user to adjust the meditation sessions to match their availability and capacity. Thus, the main aim of the present study is to examine if Headspace, a leading app-based mindfulness meditation training platform, is efficacious in reducing stress. In an attempt to clarify the prior literature, we examine different dimensions of stress, with assessments of stress measured repeatedly throughout an 8-week training period.

## Using mHealth apps to reduce stress

Most US adults report having a smartphone, and there is a growing preference from consumers for digital psychotherapeutic interventions.^3^ A digital mindfulness intervention overcomes challenges associated with traditional in-person training by facilitating broader availability, anonymity, and accessibility.^4^ Research on the effects of app-based mindfulness training is rapidly growing, with research indicating the potential for benefits to subjective well-being and mental health outcomes.^5^

The current study tests the effect of the app Headspace (Headspace, Santa Monica, CA, USA), a digital mindfulness program. Headspace is a free downloadable app (with additional content behind a paywall) that has preloaded mindfulness-based programs ranging from guided meditations to open-ended meditations with a high degree of specialization, such as targeting stress. Research on the efficacy of Headspace (and other app-based mindfulness programs) to impact stress is mixed. Some initial work has shown Headspace reduces stress among various populations.^6–9^ Yet, other research has shown no effects on self-reported stress,^10,11^ even when participants reported high levels of stress at the beginning of the study and showed high compliance with meditation.^10^ Indeed, a systematic review of randomized controlled trials testing the efficacy of app-based mindfulness programs to reduce distress found that 57% of Headspace studies demonstrated positive effects, including reductions in depression, anxiety, stress, and other measures of well-being.^2^ For stress specifically, however, only 40% of studies showed a positive effect.^2^ Thus, although Headspace can have a positive impact, there is nuance to these findings. We propose that this competing set of results might be a product of studies focusing on measuring effects only at the beginning and end of the intervention and being limited in the assessment of stress dimensions.

## Timing of assessment

Programs teaching mindfulness have historically been 8–12 weeks.^12^ Yet, it is unclear if this time commitment is needed to achieve benefits. One study assessed perceived stress scores via a survey each week during an eight-week-long MBSR program,^13^ finding reductions in stress starting at week four. Other research on dose–response with mindfulness finds no relationship between the length of the mindfulness intervention and psychological benefits,^14^ or that even a single mindfulness episode could have protective benefits for cardiovascular and emotional responses.^15^ Considering time demands represent a barrier to participation,^14^ it is critical to establish how long an intervention must be for it to produce beneficial effects.

A single mindfulness session has been indicated to produce benefits, although these are characterized as short-term rather than long-term benefits.^16^ Even when initial effects are observed, additional gains can still be achieved. Mindfulness interventions train practitioners to attend to their internal and external experiences with an attitude of nonjudgment and openness.^17^ The process of mindfulness is thought to bring about a fundamental shift in perspective and undo automatic maladaptive habitual patterns.^18^ Some of these changes may happen quickly, with others taking longer. Baer and colleagues found continued improvements in perceived stress as well as mindfulness and related skills each week during an 8-week MBSR program.^13^ In research on the current sample, changes in the mindfulness skills of attention, acceptance, and non-reactivity were observed within two weeks and continued through the eight weeks of meditating using Headspace.^19^ Findings like these highlight the dynamic effects of meditation with benefits emerging on different time scales depending on the outcomes.

## Type of stress

In studying the effects of interventions on stress, it is essential to recognize that stress is a multidimensional construct.^20^ The dimensions of stress include subjective assessments of general stress levels, the reporting of stress events and appraisals of how negative those events are, the perceived resources available to cope, and the extent to which stress is mentally represented in one’s mind as perseverative cognitions (e.g., rumination, worry). Stress research testing these dimensions has suggested they have unique relationships with well-being.^21^ Yet, app-based mindfulness research rarely defines which dimension of stress is being analyzed. The few studies that have been conducted have typically measured a single dimension, relying on measures that we interpret as capturing appraisals of perceived stress,^6,9,22^ the inability to cope with stress,^7^ or more generic ratings of distress that include measures of feeling stressed, anxious, and depressed.^8^ Also, the length of the app-based mindfulness meditation practice varies in each study, such as being ten minutes of practice for ten days,^6–8^ for about 4 weeks,^6,9^ or eight weeks.^22^ This length of time is important when considering this practice and may potentially impact each dimension differently.

Given that stress consists of related yet distinct dimensions, it is plausible that mindfulness meditation would not affect each stress dimension similarly. Mindfulness produces changes in decentering,^18^ acceptance,^23^ and nonreactivity to inner experience,^24,25^ each of which may alter how a person perceives the purpose of stress. For example, decentering involves a fundamental shift in perspective, allowing a person to step outside of their immediate experience and view this experience with greater clarity and objectivity.^18^ Therefore, we might expect that decentering could lower one’s subjective stress they feel at any moment. Acceptance is the ability to observe experiences with an attitude of nonjudgment and openness.^17^ As such, this may help a meditator process unwanted and unpleasant thoughts and reduce engagement and reactivity to them, thus lowering perseverative cognitions. For perceived coping and appraisal of stressors, the impact of mindfulness may be indirect. Mindfulness meditation focuses on cultivating acceptance of momentary experiences rather than attempting to alter one’s reaction to or judgment of an event. As such, app-based mindfulness practices may not provide explicit training in improving self-efficacy when faced with specific stressors on their own (especially compared to in-person training, such as MBSR, in which meditators have a chance to discuss the application of knowledge with their trainer). We expect this to be particularly true for our sample of novice meditators who may not be able to extend the learned skills of decentering and acceptance to novel situations.

## The present study

The present study examined if Headspace reduces stress among novice meditators, focusing on when effects emerge during the 8-week intervention. In examining the effects of stress, we tested different dimensions of stress over time. Stress dimensions were assessed using four-day bursts of ecological momentary assessment (EMA) at baseline and then weeks 2, 5, and 8 post-enrollment, with five assessments each day. EMA is a method that involves the repeated sampling of subjects’ current behaviors and experiences in real-time in the subjects’ natural environment.^26^ The power of EMA is it can minimize recall bias, maximize ecological validity, and allow the study of micro-processes that influence behavior in real-world contexts.^27^ EMA has previously been used to track how mindfulness increases during meditation practice compared to daily living,^28^ and has shown the ability to track changes in mindful states.^29^

Based on prior work demonstrating relatively quick changes in mechanisms after the onset of training,^13,19^ we predicted changes in stress would also be observed by the second week of training. However, given the targeted mechanisms of mindfulness meditation training, and Headspace specifically, we expected changes to occur in the stress dimensions of subjective stress and perseverative cognitions, but anticipated the possibility for minimal or null effects for stress appraisals and/or perceived coping.

## Method

### Study design

We employed a randomized controlled trial comparing mindfulness meditation through the app Headspace to an inactive, waitlist control condition. The main manipulation was being randomized to have access to the Headspace app, as well as simple instructions and encouragement to engage in meditation. Stress was measured at baseline, during the 8-week intervention at weeks 2 and 5 post-randomization, and post-intervention at week 8. Each measurement time point (baseline, week 2, week 5, week 8) consisted of four consecutive days of EMA with five assessments per day.

### Participants

Participants were staff workers of the University of California, Merced, a public university located in Merced, California. Participants were excluded if they were faculty or students, were not employees at the university, younger than 18 years of age, were not fluent in English, did not have access to a smartphone with internet access every day, or were experienced meditators, defined as having participated in a sitting meditation practice more than twice a week (for 10 minutes or greater each episode) over the last three months. After visiting a website to learn more about the study, interested participants completed an online survey to assess eligibility criteria.

**Figure 1.**
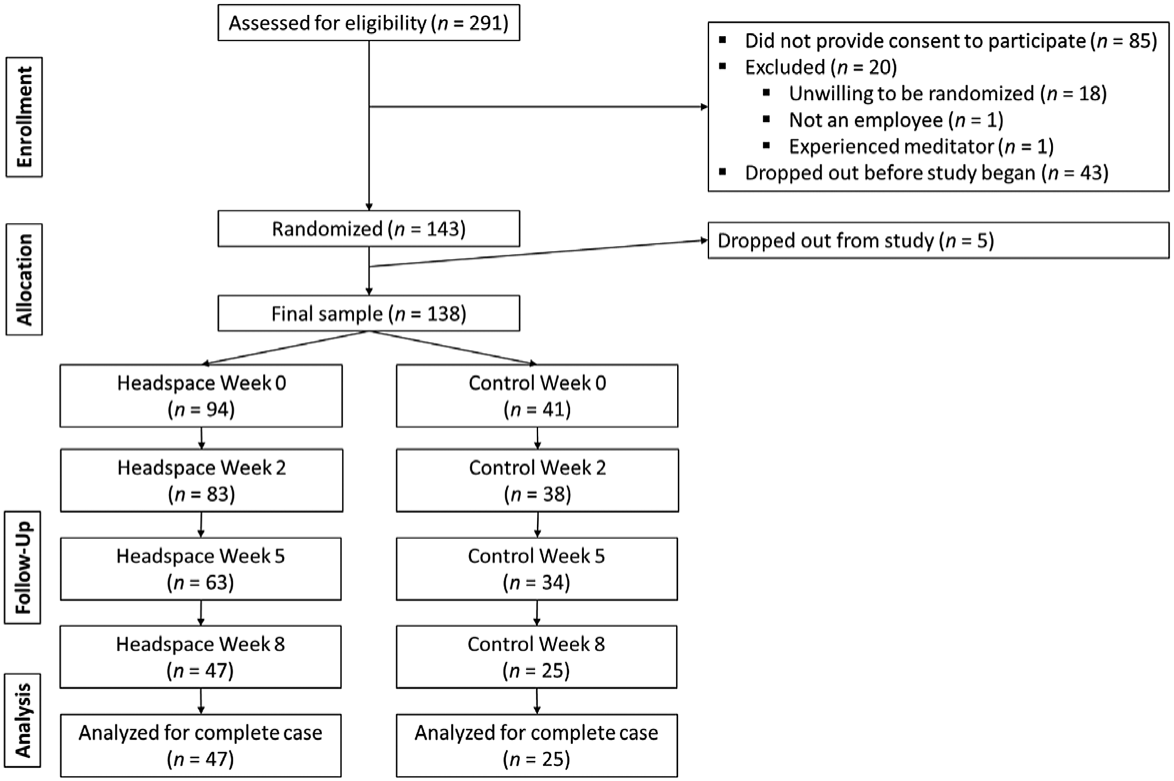
CONSORT diagram.

As can be seen in Figure 1, a sample of 291 employees was assessed for eligibility, with 143 participants randomized. Of this, the final sample consisted of 138 employees who completed at least one assessment at baseline. Demographics for the sample can be seen in Table 1. Participants in the final sample were between the ages of 21 and 65 years old and averaged middle-aged, self-identified on a race/ethnicity question as primarily White or Hispanic, were majority female, and had a range of education, including had either less than a B.A., had a B.A., or had a professional degree. Those who completed the study did not differ from those who dropped out in terms of baseline stress (*M *= 19.10, *SD *= 5.74 versus *M *= 19.11, *SD *= 5.87, respectively; *t*(136) = 0.02, *P = *.99), age (*M *= 38.43, *SD *= 11.43 versus *M *= 37.92, *SD *= 10.46, respectively; *t*(136) = -0.27, *P = *.79), proportions identifying as Hispanic (26.39% versus 28.79%, respectively; χ^2^ = 0.10, *P = *.75) or White (33.33% versus 26.81%, respectively; χ^2^ = 0.88, *P = *.35), proportion female (68.18% versus 81.94%, respectively; χ^2^ = 3.51, *P = *.06), nor proportion with less than a B.A. (13.04% versus 15.28%, respectively; χ^2^ = 0.14, *P = *.70), with a B.A. (31.88% versus 34.72%, respectively; χ^2^ = 0.13, *P = *.72), or with a professional degree (55.07% versus 50.00%, respectively; χ^2^ = 0.36, *P = *.55).

**Table 1:** | Demographics and stress levels of participants at baseline.

Variable	Statistic	Headspace Group	Control Group	Overall
Age	*M*	36.91	41.12	38.19
	*SD*	10.84	10.71	10.94
Race and ethnicity^1^				
American Indian or Alask Native	*n* (%)	1 (1.04%)	2 (4.76%)	3 (2.17%)
Asian	*n* (%)	7 (7.29%)	4 (9.52%)	11 (7.97%)
Black	*n* (%)	3 (3.13%)	1 (2.38%)	4 (2.90%)
Hispanic	*n* (%)	30 (31.25%)	8 (19.05%)	38 (27.54%)
Pacific Islander	*n* (%)	2 (2.08%)	1 (2.28%)	3 (2.17%)
White	*n* (%)	57 (59.38%)	26 (61.90%)	83 (60.14%)
Gender				
Female	*n* (%)	74 (77.08%)	30 (71.43%)	104 (75.36%)
Male	*n* (%)	22 (22.92%)	12 (28.57%)	34 (24.64%)
Education				
less than B.A.	*n* (%)	12 (12.50%)	8 (19.05%)	20 (14.49%)
B.A.	*n* (%)	29 (30.21%)	17 (40.48%)	46 (33.33%)
Professional degree	*n* (%)	54 (56.25%)	17 (40.48%)	71 (51.45%)
Missing	*n* (%)	1 (1.04%)	0 (0.00%)	1 (0.72%)
Perceived stress (baseline)	*M*	19.56	18.07	19.11
	*SD*	5.93	5.36	5.78

Participants could select all race and ethnicity categories that apply.

### Materials

#### Baseline measures

During baseline, participants completed demographic information about their gender (coded as 0 = male, 1 = female), age (in years), race and ethnicity (selecting all that apply from the following options: American Indian or Alaska Native, Asian, Black or African American, Hispanic or Latino, Native Hawaiian or Pacific Islander, White), education, and other measures not relevant to the present study. For analyses, ethnicity was coded as either non-Hispanic (0) or Hispanic (1). Education was dummy coded as either participants with less than a B.A. or with a B.A. (or 4-year degree), with these two variables compared to those with a professional degree (including a Master’s or Doctorate).

Additionally, to control for initial levels of perceived stress, participants completed the 10-item Perceived Stress Scale.^30^ Using this scale, participants report the frequency of thoughts and feelings related to being unable to cope with stressors over the past month (e.g., “In the last month, how often have you felt that you were unable to control the important things in your life?”). Participants responded on a 0 (*never*) to 4 (*very often*) scale. Items were averaged together such that higher values indicate more perceived stress (*α* = .87).

#### EMA measures

Subjective stress, stressor appraisals, perceived coping, and perseverative cognitions were assessed using EMA, based on past research on how to measure stress in everyday life.^31^ To assess subjective stress, participants rated their level of stress in the moment on a 0 (*not at all*) to 10 (*extremely*) sliding scale using the following item: “How stressed do you currently feel?” Next, to assess stressor appraisals participants were asked about the occurrence of stressful events with the following yes or no item: “Since the last prompt, has anything stressful occurred?” If participants reported that an event occurred, they were asked to rate how stressful the event was on a 0 (*not at all*) to 10 (*extremely*) sliding scale using the following item: “If yes, how stressful was it?”

Next, participants were asked about perceived coping. Participants responded to the following four items, adapted from the short-form Perceived Stress Scale, on a sliding scale from 0 (*not at all*) to 10 (*extremely*): “Right now, do you feel you could control important things?”; “Right now, do you feel confident in your ability to handle problems?”; “Right now, do you feel things are going your way?”; and “Right now, do you feel difficulties piling up so that you cannot overcome them?” Items were adapted to assess how one was feeling at the present moment. Items at each assessment were averaged such that higher values indicated more stress coping at that moment. Across all assessments, the items indicated a strong reliability to detect within-subjects differences in change over time, *R*_*C*_ = 0.73.^32^

To assess perseverative cognitions, participants were asked the following questions on a sliding scale from 0 (*not at all*) to 10 (*extremely*): “Right now, how much are you ruminating or thinking about past events that were not well resolved?”; “Right now, how much are you worrying?”; “Since the last survey, how much has a negative thought kept popping into your mind?”; “Since the last survey, how much have you had the same negative thought repeating in your head over and over again?”; and “Since the last survey, how much have you been unable to get a negative thought out of your mind?” A composite score of the items in each assessment was created with higher values indicating more engagement in perseverative cognitions at that moment. Across all assessments, the items indicated a strong reliability to detect within-subjects differences in change over time, *R*_*C*_ = 0.88.

#### Headspace

The intervention was delivered through the commercially available mindfulness meditation app Headspace, which has been widely used in previous intervention studies.^33,34^ Headspace provides guided and unguided meditation practices, with instructions delivered through short, animated training videos. The intervention group was instructed to start meditating using the Basics pack. This pack is designed as an introduction to mindfulness meditation and for participants to get familiar with the Headspace teaching style. It presents the concept of mindfulness and encourages participants to pay attention to breathing and notice patterns of mind-wandering and thoughts. Once participants completed Basics 1, 2, and 3 (10 episodes each), they were instructed to move on to the Stress pack, which lasted for 30 episodes. This pack was more specialized, combining visualization and body scanning to help users learn to accept their emotions and pay close attention to the present moment.

For each episode, participants were encouraged to complete the meditation in a quiet place where they would not be distracted. They were instructed to use the app to meditate for 10 minutes a day for 8 weeks. We chose 10 minutes based on prior work showing that just 10 days of practicing mindfulness 10 minutes a day successfully reduced stress, negative affect, and well-being among a range of sample types.^6–9^ Participants were given the choice of when and how they performed their meditation (e.g., they decided when in the day they would meditate).

### Procedure

All study procedures were approved by the local Institutional Review Board. The study was registered on ClinicalTrials.gov (NCT03652168). There were no deviations from the approved protocol and the analyses presented are those from the primary set of outcomes proposed. All data were collected between October 2018 and August 2019. Participants were recruited through posted flyers on campus, presentations at departmental and university staff assembly meetings, and word-of-mouth referrals. The study was advertised to participants through a series of questions including asking whether the participant has wondered about the benefits of mindfulness meditation, looked for ways to reduce stress in the workplace, wondered if meditation could alleviate levels of daily stress, and/or looked for ways to increase centeredness and happiness. Interested participants were directed to a secure website in which they read more about the study and were directed to a screener delivered through Qualtrics (Provo, UT). Eligible participants were then sent a survey to complete that included informed consent, demographics, and baseline stress levels.

Once the survey was completed, participants attended an in-lab orientation lasting about 60 minutes. Each orientation took place on a Monday, Tuesday, or Wednesday morning. This orientation introduced participants to the EMA procedure. First, they downloaded the RealLife Exp application (LifeData, Marion, IN) on their smartphone and loaded the survey. Participants were guided through a startup survey that demonstrated how to navigate the application and practiced answering the EMA questions. After practicing, participants were instructed that when a survey was ready for completion they would receive a push notification, as well as see an indicator in the app of the available survey. Surveys were programmed to randomly occur once in each of the following windows: 8:00-10:00am, 10:30am-12:30pm, 1:00-3:00pm, 3:30-5:30pm, and 6:00-8:00pm. Participants had up to one hour to complete a survey with a reminder notification occurring 20 minutes after the initial prompt. Surveys took between 3 and 4 minutes to complete on average.

There were four consecutive days of data collected via EMA. All EMA data were collected on Wednesday through Saturday to ensure relative commonality across participants and to ensure both working and non-working days. Participants completed an initial four-day burst (labeled baseline), and then completed additional four-day bursts two (week 2), five (week 5), and 8 (week 8) weeks after the first day of EMA data. Upon completion of (or withdrawal from) the study, participants were paid $15 for each week, and were awarded an additional $20 if they completed at least 80% of surveys across all weeks.

After the baseline four days of EMA data collection, participants were randomized into the Headspace or control condition. Allocation to condition occurred at a 2:1 ratio with more people in the Headspace condition, to account for potential dropout. Participants in the Headspace condition were sent an email containing download instructions for the Headspace application and a code to enter that would grant 12 months of access to Headspace. Participants were instructed on how to use Headspace. To ensure onboarding, we tracked downloads and initial use of the app, ensuring all participants completed their first mindfulness training within the first-week post-randomization. For the control group, participants were emailed that they were on the waitlist. As part of being on the waitlist, they were asked not to participate in any mindfulness activities like yoga or meditation during the 4-month time period. They were told that after this waiting period, they would be given all the same information and access to Headspace as the Headspace condition. All participants were sent weekly text messages or emails (depending on their preferred contact method) with encouragement. For the Headspace group, these messages were generic in nature either describing tips to incorporate meditation into their life (e.g., “You’re on the way to becoming a meditator! Having trouble meditating daily? Be patient with yourself. Try setting daily reminders on Headspace under settings.”; “Like any skill, the best way to improve is to practice! It can take 21 days to form a habit. Make a commitment to meditate & watch yourself progress!”) or to remind them about the study details (e.g., “Congratulations! You’re almost halfway through the study! Be on the lookout for the midway questionnaire. Take the journey one day at a time and do your best.”). For the control group, these text messages were about the study assessment (e.g., “Congratulations, you’ve made it through your first week in the Stress Free UC Study! Your participation is very valuable to us. Questions? Let us know.”).

For the control group, there were 619/820 (75.49%) completed EMA surveys for baseline, 610/760 (80.26%) for week 2, 458/680 (67.35%) for week 5, and 342/500 (68.40%) for week 8, for an overall compliance rate of 73.51%. For the Headspace group, there were 1467/1880 (78.03%) completed EMA surveys for baseline, 1250/1660 (75.30%) for week 2, 914/1260 (72.54%) for week 5, and 600/940 (63.83%) for week 8, for an overall compliance rate of 73.71%.

### Analytic plan

We used two-level models with momentary observations nested within participants. Models were tested using the proc mixed command in SAS v.9.4. Our models tested for fixed effects of whether time (baseline, week 2, week 5, week 8), being in the Headspace condition (0 = control group, 1 = Headspace group), and their interaction predicted stress levels. Time was modeled as a class variable such that weeks 2, 5, and 8 were compared to the baseline data. In the data presented in the table, the Time variables represent the effects over weeks for those not in the Headspace condition; in turn, the interaction terms represent the effects over weeks for those in the Headspace condition compared to the control condition. After checking for outliers, each stress variable was tested in a separate model.

In line with recommendations to enhance power, we included covariates in the model.^35,36^ At the between-person level, models controlled for gender, age, ethnicity, education, and baseline stress levels as measured by the Perceived Stress Scale. At the within-person level, models controlled for day of week as either a non-weekend (0) or weekend (1) day, day of the study within week (ranging from 1 to 4), and time of day in hours. Random intercepts at the person level were modeled to account for the possibility of differing stress levels for each person at baseline. A pseudo *R*^2^ was calculated as an indicator of effect size by estimating a predicted value for each datapoint and then correlating this estimate with the observed value.^37^

Two sets of models were run in line with best practices to test for effects in randomized controlled trials.^38^ First, we performed intention-to-treat (ITT) analyses in which those randomized to condition were assumed to complete the intervention material as conducted. Second, to address potential attrition effects, only those who completed data collection through week 8 were included as part of a complete case (CC) analysis. Following recommendations,^39^ restricted maximum likelihood estimation was used to handle missing data as it estimates probable values rather than imputing them, which can be a problematic approach with missingness not at random.

## Results

### Descriptive statistics

At the start of the study, participants reported an average stress level of 19.11 (*SD *= 5.78) on the Perceived Stress Scale, which places participants in the moderately stressed category. Demographics of participants by condition can be found in Table 1. Participants in the control condition were slightly older than participants in the Headspace condition, *t*(136) = 2.11, *P = *.037. Those in the control condition did not differ from those in the Headspace condition in terms of baseline stress, *t*(136) = -1.39, *P = *.17, proportion identifying as Hispanic, χ^2^ = 2.18, *P = *.14 or as White, χ^2^ = 0.08, *P = *.78, proportion female, χ^2^ = 0.28, *P = *.48, nor proportion with less than a B.A., χ^2^ = 0.96, *P = *.33, with a B.A., χ^2^ = 1.29, *P = *.26, or with a professional degree, χ^2^ = 3.12, *P = *.08.

### Headspace by time analyses

**Figure 2.**
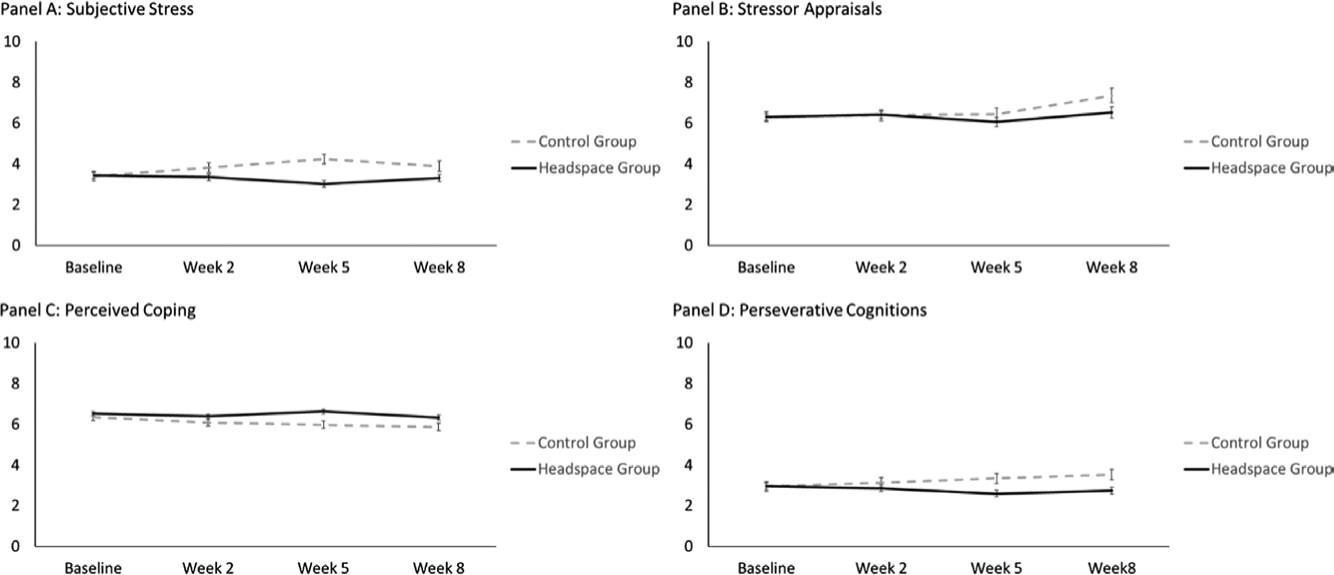
Stress levels as a function of headspace and time.

We tested whether participants in the Headspace and control conditions had less stress at weeks 2, 5, and 8 than at baseline. Data for each week was assessed using EMA with up to 20 assessments per week per person. Thus, results reveal whether stress levels within each stress dimension are different across all reports in a week compared to all baseline EMA reports. Below we describe the ITT analyses as they show a similar pattern of effects to the complete case analyses. Both the ITT and complete cases analyses are presented in Table 2. To help visualize these effects, we estimated least squares means (and standard errors) for time by condition for each of the stress outcomes. These values are plotted in Figure 2.

**Table 2:** **|** Unstandardized beta coefficients (standard errors) for the effect of headspace and time on stress dimensions.

	Subjective stress		Stressor appraisals		Perceived coping		Perseverative cognitions	
	ITT	CC	ITT	CC	ITT	CC	ITT	CC
*Random effects*								
Intercept (Person)	**1.86** **(0.25)**	**1.85** **(0.34)**	**0.89** **(0.18)**	**0.95** **(0.24)**	**1.03** **(0.13)**	**1.11** **(0.20)**	**2.12** **(0.27)**	**2.02** **(0.37)**
Residual	**4.40** **(0.08)**	**4.34** **(0.10)**	**3.32** **(0.16)**	**3.22** **(0.20)**	**1.55** **(0.03)**	**1.57** **(0.03)**	**2.50** **(0.05)**	**2.47** **(0.05)**
*Fixed effects*								
Intercept	0.83 (0.91)	0.31 (1.17)	**5.02** **(0.86)**	**5.01** **(1.13)**	**8.77** **(0.67)**	**9.52** **(0.89)**	0.03 (0.95)	-1.04 (1.21)
Female	−0.005 (0.29)	−0.32 (0.44)	0.34 (0.26)	0.10 (0.38)	0.09 (0.21)	0.40 (0.34)	0.12 (0.30)	0.23 (0.46)
Age	0.001 (0.01)	0.01 (0.02)	−0.004 (0.01)	−0.02 (0.01)	0.02 (0.01)	0.004 (0.01)	−0.003 (0.01)	0.01 (0.02)
Hispanic	−0.34 (0.29)	−0.55 (0.42)	0.50 (0.26)	0.17 (0.38)	0.27 (0.21)	0.44 (0.32)	−0.29 (0.30)	−0.40 (0.43)
Less than BA	−0.18 (0.37)	0.18 (0.51)	0.41 (0.34)	0.50 (0.46)	−0.04 (0.27)	−0.24 (0.39)	0.21 (0.39)	0.47 (0.52)
BA	−0.16 (0.28)	0.16 (0.37)	0.07 (0.25)	0.28 (0.33)	0.06 (0.20)	−0.18 (0.29)	0.11 (0.29)	0.24 (0.39)
Baseline stress	**1.40** **(0.21)**	**1.55** **(0.30)**	**0.47** **(0.20)**	**0.71** **(0.29)**	**−1.23** **(0.16)**	**−1.36** **(0.23)**	**1.36** **(0.22)**	**1.55** **(0.32)**
Weekend	**−0.74** **(0.06)**	**−0.85** **(0.08)**	−0.05 (0.17)	−0.08 (0.21)	**0.27** **(0.04)**	**0.31** **(0.05)**	**−0.39** **(0.05)**	**−0.40** **(0.06)**
Study day	**−0.11** **(0.02)**	**−0.07** **(0.03)**	−0.05 (0.05)	−0.04 (0.06)	**0.05** **(0.01)**	0.03 (0.02)	**−0.11** **(0.02)**	**−0.09** **(0.02)**
Time of day	**−0.05** **(0.01)**	**−0.06** **(0.01)**	−0.02 (0.02)	−0.02 (0.02)	**0.01** **(0.005)**	0.01 (0.01)	**−0.03** **(0.01)**	**−0.03** **(0.01)**
Week 2	**0.43** **(0.12)**	**0.38** **(0.15)**	0.08 (0.28)	−0.08 (0.36)	**−0.26** **(0.07)**	**−0.27** **(0.09)**	**0.19** **(0.09)**	0.15 (0.11)
Week 5	**0.85** **(0.14)**	**0.76** **(0.15)**	0.12 (0.33)	0.29 (0.40)	**−0.37** **(0.08)**	**−0.38** **(0.09)**	**0.39** **(0.10)**	**0.44** **(0.12)**
Week 8	**0.50** **(0.15)**	**0.46** **(0.15)**	**1.04** **(0.37)**	**1.03** **(0.39)**	**−0.48** **(0.09)**	**−0.49** **(0.09)**	**0.59** **(0.11)**	**0.59** **(0.12)**
Headspace	0.05 (0.29)	−0.07 (0.39)	−0.01 (0.29)	−0.09 (0.39)	0.17 (0.21)	0.17 (0.29)	0.002 (0.29)	−0.36 (0.40)
Week 2 × Headspace	**−0.52** **(0.15)**	**−0.59** **(0.19)**	0.03 (0.34)	0.004 (0.43)	0.13 (0.09)	0.09 (0.11)	**−0.28** **(0.11)**	−0.27 (0.14)
Week 5 × headspace	**−1.26** **(0.16)**	**−1.31** **(0.19)**	−0.36 (0.40)	−0.19 (0.49)	**0.48** **(0.10)**	**0.53** **(0.12)**	**−0.76** **(0.12)**	**−0.85** **(0.15)**
Week 8 × headspace	**−0.62** **(0.18)**	**−0.66** **(0.19)**	−0.81 (0.46)	−0.78 (0.48)	**0.39** **(0.11)**	**0.29** **(0.12)**	**−0.81** **(0.14)**	**−0.82** **(0.15)**
*Model statistics*								
Pseudo *r*^*2*^	0.12	0.15	0.05	0.05	0.18	0.20	0.12	0.16

Coefficients in **bold** significant at *P* < .05. Weeks 2, 5, and 8 are compared to baseline. ITT indicates intention-to-treat analyses. CC indicates complete case analyses.

For subjective stress, those in the control condition, compared to baseline, reported *more* stress at weeks 2, 5, and 8 (*P*s < .001). There was a significant interaction of time by condition such that those in the Headspace condition versus the control group reported *less* stress at weeks 2, 5, and 8 compared to baseline (*P*s < .001).

For stressor appraisals, those in the control condition reported stressors as *more* stressful at week 8 compared to baseline (*P = *.005), but no difference at week 2 (*P = *.78) nor week 5 (*P = *.71). For the Headspace condition, there was not a significant interaction effect of time. There were no differences in stressor appraisals at weeks 2 (*P = *.93), 5 (*P = *.36), nor 8 (*P = *.08) compared to baseline.

For perceived coping, those in the control condition reported *less* coping at weeks 2, 5, and 8 compared to baseline (*P*s* < *.001). There was a significant interaction of time by condition such that those in the Headspace condition versus the control group did not report differences in coping at week 2 compared to baseline (*P = *.16), but reported *more* coping by week 5 (*P < *.001) and week 8 (*P = *.009).

For perseverative cognitions, those in the control condition reported *more* engagement in perseverative cognitions at week 2 (*P = *.049), and weeks 5 and 8 (*P*s < .001), compared to baseline. There was a significant interaction of time by condition such that those in the Headspace condition versus the control group reported *less* engagement in perseverative cognitions at week 2 (*P* = .012), and weeks 5 and 8 (*P*s* < *.001), compared to baseline.

### Exploratory analyses

We tested if there was a quadratic effect of time by condition. We ran the same models as above, except we tested time as a linear variable, included a quadratic term for time, and then included interactions of both the linear time by condition and quadratic time by condition. Results support the visualization in Figure 2 which appears to show a small dipping effect for subjective stress and perceived coping after initial gains. Specifically, for subjective stress, there was both a significant interaction for time by condition (*b *= −0.46, *SE* = 0.07, *P < *.001), as well as a quadratic effect for time by condition (*b *= 0.05, *SE* = 0.01, *P < *.001). A similar pattern was found for perceived coping with both significant effects for time by condition (*b *= 0.15, *SE* = 0.04, *P < *.001) and quadratic time by condition (*b *= −0.01, *SE* = 0.01, *P = *.018). For perseverative cognitions, only the linear (*b *= −0.21, *SE* = 0.05, *P < *.001), and not the quadratic (*b *= 0.01, *SE* = 0.01, *P = *.07), interaction term was significant. Neither the linear (*b *= 0.02, *SE* = 0.17, *P = *.89) nor quadratic (*b *= −0.01, *SE* = 0.02, *P = *.44) interaction of time by condition was significant for stressor appraisals.

## Discussion

The purpose of the study was to examine whether the app-based mindfulness tool Headspace was effective in reducing stress for staff members of a university campus who were novice meditators over an 8-week intervention. We moved beyond a traditional pre-post design by conducting repeated assessments of stress throughout the intervention. We also measured different dimensions of stress. Thus, our goals were to examine the trajectory of change among stress dimensions examining when effects emerge and if effects are maintained. Overall, we observed significant reductions for the Headspace group in subjective stress and perseverative cognitions by week 2 that persisted through the end of the intervention, significant increases in perceived coping by week 5 that persisted, but no changes in appraisals of stress events. These results suggest the importance of measuring stress in a multidimensional way and may help explain why inconsistent results have been observed in past studies.^2^

Improvements in subjective stress and perseverative cognitions were observed quickly. This type of rapid response to meditation, especially for novice meditators, is not uncommon. For example, in other work, 10 days of in-person meditation in novice meditators decreased rumination and negative affect.^40^ These quick effects may be due to how mindfulness interventions work. The content of the initial Headspace training is to become consciously aware of one’s cognitions while learning how to acknowledge these thoughts and feelings without needing to dwell on or react to those cognitions. Past research with this sample,^19^ and other in-person training,^13^ showed that participants can develop the ability to be more nonreactive to negative stimuli within two weeks of meditating. Given that these skills help a person to distance themselves from negative stimuli, including stressful thoughts that are often internally driven,^31^ it makes sense that subjective stress and perseverative cognitions demonstrated the earliest impacts.

The effects for perceived coping did not emerge until week 5, which may be due to the participants encountering a learning period when engaging in mindfulness meditation. This supports predictions that these effects may be indirect and take longer to develop as skill acquisition models would predict.^41^ Perceived coping may involve more proactive understanding of one’s ability to act in a novel and unexpected situation. For these novice meditators, translating their understanding of the skills to imagine how one could cope with hypothetical stressors likely represented a difficult task. Also, the content related to coping with stress was not explicitly addressed in the Basic pack, but instead was a skill introduced in the Stress pack that was made available for training after basic mindfulness skills were learned.

Headspace did not affect stress appraisals of reported stressors. This finding may appear to contradict prior research showing that having higher levels of mindfulness helped students appraise an exam as less threatening.^42^ Yet, these appraisals appear to be for future events and may function more like worry rather than a reaction to a stressor event that is ongoing. This null finding was anticipated as a possibility because the Headspace training does not necessarily train a person to be more efficacious when dealing with specific events; instead, mindfulness focuses on acceptance of the momentary experience as is rather than interpreting it as positive or negative and/or taking direct action against the stressor. It is possible that this is an even more difficult task to learn than perceived coping, as the stressor moment may demand action and cause perturbations to psychological and physiological systems that disrupt clear thinking.

## Implications

We found evidence that an app-based mindfulness meditation training reduced momentary stress perceptions and perseverative cognitions. These findings are important as this delivery method may increase intervention access for people who have historically been excluded and who currently encounter barriers to intervention participation.^43^ The apps may also foster anonymity to address the barrier of mental health stigma,^44–46^ which is often perpetuated by cultural attitudes.^47^ Moreover, the convenience of the intervention on a personal device gives the individual the ability to access the intervention when it might be needed most.^48^ Apps may also be a crucial intervention tool for regions in which mental health services and primary prevention are limited, including the region where the current study was conducted. Thus, app-based interventions could be valuable for individuals from underserved groups, but it is necessary to identify the features that are most effective at promoting and maintaining use to ensure changes are appropriate for these groups.

As noted, the results for the various dimensions of stress peaked by week 5. This may be due to participants encountering “learning stages” where practicing mindfulness skills became more difficult and potentially requires more training. Mindfulness theory generally proposes that the skill of attention must be practiced and built prior to engaging in the skill of acceptance.^49^ As novice meditators, the participants were likely inexperienced with practicing and combining these skills, especially in real-life situations. The shift from practicing attention to learning and incorporating acceptance may have been particularly challenging, and thus impacted when outcomes changed and the maintenance of the change. Encountering challenges and ineffectiveness represent vulnerable moments during an intervention that may lead participants to lessen engagement or fully withdraw from training.^50^ Practitioners of mindfulness may wish to inform trainees that skills develop at different rates to help identify if and when training is working.

The findings of the present study also suggest the context in which an app-based intervention is implemented might be important to consider. Headspace was implemented in the workplace, and although workplace outcomes were not directly examined, the control group, unlike the intervention group, displayed an increase in stress over the assessment period. This might indicate the larger employment context was already a stressful place. Relatedly, within a certain context, there may be specific outcomes and situations for which the intervention is more or less effective.^51^ For example, mindfulness might be effective when an individual is experiencing burnout, but may not be as useful, or is possibly hurtful, when dealing with work-home conflict.^52^ Thus, it could be necessary to consider moderators, such as the source of stress, type of job, or position, when testing the effectiveness of an app-based mindfulness intervention in the workplace.

## Limitations and future directions

We did not have continuous usage statistics for Headspace. We intended to have end-of-day reports to track how Headspace was being used. Unfortunately, due to technical errors, these reports were not delivered to participants consistently to be usable. Thus, as with every intervention, there is the chance that results could be due to the placebo effect. Yet if a placebo effect were the prime driver of the findings, we would expect all the stress variables to be similarly affected at similar times. Instead, our pattern of findings of changes in subjective stress and perseverative cognitions lines up with prior work with this sample showing attention and acceptance changing by week 2.^19^ More so, in this prior work, there were no changes in other mechanisms not explicitly taught by Headspace, such as self-compassion, again suggesting the lack of a generalized effect.

There was an unexpected weakening of effects by week 8 perhaps indicating a lack of maintenance of initial gains. It is possible that users experienced clear improvements by week 5 such that they became content and either discontinued meditating or significantly decreased engagement. If so, the intention-to-treat analyses, which assumed everyone in the Headspace group was actively using the app, may have actually *underestimated* the effect of Headspace that we observed. Thus, there is a need to develop better ways to track usage for interventions that are done daily.

Finally, the rate of participant attrition from pre- to post-intervention was ~50%. Unfortunately for the field, this is not uncommon for app-based programs.^53^ One possibility of the high attrition rate is that the observed effect sizes are inflated. However, we observed similar patterns of results with the intention-to-treat and complete case analyses, suggesting it is unlikely that only those benefiting most remained in the study. Instead, it becomes important to consider that the dropout rate seemed to display a sharp increase after week 5. This roughly corresponds to when participants should have completed the three Basic Pack sessions of 10 units each. It is possible that at this point participants were unsure of what content to migrate to next or felt they had completed all necessary training. This possibility highlights the value of developing ways to maintain engagement. Existing research on app-based interventions suggests engagement reminders, provider support, and personalization might be effective.^48^ Research should also focus on *who* are the people dropping out of these interventions and *why* this occurs. Identifying whether people who drop out share similar traits (e.g., low trait mindfulness) or face similar barriers (e.g., culturally unrelatable material) could be useful when adapting app-based mindfulness interventions to meet the needs of individuals from underserved groups and encourage engagement.

## Conclusions

The current study joins the larger field of meditation research by showing that an mHealth app can reduce a person’s stress. The Headspace app proved to be an effective tool to improve stress in a novice meditator and non-clinical population in as little as two weeks, but the effects of stress did vary by the dimension of stress that was assessed. It also uncovered areas where more investigation is warranted, including investigating how frequency and duration of sessions interact to form the optimal intervention.
